# Ultrasound-guided suprainguinal fascia iliaca block versus lumbar erector spinae block for oncologic thigh surgery

**DOI:** 10.1097/PR9.0000000000001334

**Published:** 2025-08-27

**Authors:** Ahmed Salah Abdelgalil, Hamed Sayed Ashour, Ayman Sharawy Abdelrahman, Ahmed Fahmy Ahmed, Fatma Hanafi Mahmoud, Mohamed El Sayed Hassan, Khaled Ali El Samahy, Sayed Mahmoud Abed

**Affiliations:** Pain Relief and Intensive Care Unit, National Cancer Institute, Cairo University, Cairo, Egypt

**Keywords:** Ultrasound, Suprainguinal fascia iliaca block, Lumbar erector spinae plane block, Oncology, Thigh surgery

## Abstract

**Introduction:**

Effective pain management is essential for patients undergoing limb-sparing procedures as well as amputation in patients with cancer.

**Objectives:**

This study evaluated the analgesic effects of the ultrasound-guided suprainguinal fascia iliaca block (SIFIB) and the ultrasound-guided lumbar erector spinae plane block (L-ESPB) in patients who underwent oncologic thigh surgery.

**Methods:**

Seventy-five patients with thigh cancers and ASA class II, III were randomized into 3 groups: SIFIB, ESPB, and control groups. The primary outcome was opioid consumption within 24 hours. The secondary outcomes were intraoperative fentanyl consumption, time to first rescue analgesic request, pain scores, and patient satisfaction.

**Results:**

In our study, only 6 patients (24%) in the SIFIB group and 3 patients (12%) in the ESPB group required a single 3 mg dose of morphine, compared with 20 patients (80%) in the control group (*P* < 0.001). There was no significant difference between the SIFIB and ESPB groups in rescue morphine use (*P* = 0.463). Total fentanyl consumption was significantly lower in both the SIFIB and ESPB groups compared with the control group (*P* < 0.001), with no significant difference between the 2 blocks (*P* = 0.953). Time to first rescue analgesia was significantly longer in the SIFIB and ESPB groups compared with the control group (*P* < 0.001) and was similar between the block groups (*P* = 1.000). Pain scores were significantly lower and patient satisfaction significantly higher in both block groups compared with the control group (*P* = 0.042), with reduced postoperative nausea and vomiting.

**Conclusion:**

In patients undergoing oncologic thigh surgery, SIFIB and L-ESPB are comparable, effective, and safe regional analgesic alternatives.

## Abstract 2

Suprainguinal fascia iliac block and lumbar erector spinae plane block provide effective pain relief, reduce opioid use, delay rescue analgesia, and improve patient satisfaction compared with the control group in thigh cancer surgery.

## 1. Introduction

The incidence of moderate and severe pain is nearly 93.3% and 18.3%, respectively, in the first 24 hours after cancer surgery.^7^ Patients who undergo cancer surgeries may already experience chronic cancer pain before the surgery. This makes postoperative pain management more complex, as there is a background of persistent pain that needs to be carefully balanced with acute surgical pain.^9^ As most patients with cancer have prolonged opioid use for cancer pain, there may be opioid tolerance or even dependence. This can complicate postoperative pain management, as effective pain control may necessitate increased opioid dosages. Consequently, the likelihood of adverse events such as nausea, constipation, and sedation is elevated, as well as the potential for opioid-induced hyperalgesia.^21^ To mitigate these risks and effectively manage pain after oncologic surgeries, multimodal pain management strategies should incorporate regional anesthesia techniques.^33^

The suprainguinal fascia iliaca block (SIFIB) represents a well-recognized and efficacious regional anesthesia method that was described by Hebbard et al.,^15^ particularly with ultrasound (US) guidance and a proximal approach.^3^ US-guided SIFIB has confirmed enhanced analgesia and a substantial reduction in opioid requirements.^6^

Lumbar erector spinae plane block (L-ESPB) is a regional anesthesia technique intended to provide pain relief through the injection of local anesthetic within the plane separating the erector spinae muscles, which run parallel to the spine, from the underlying lumbar vertebrae and their processes.^29^ Lumbar erector spinae plane block provides postoperative pain management and improved postoperative recovery with minimal complications.^11^

The SIFIB and L-ESPB blocks were chosen because they are both promising options for pain control in lower-limb surgeries, but they differ in their anatomical approach and mechanisms of action. The SIFIB targets the femoral, lumbar, and sciatic nerves, offering broad analgesia, while the L-ESPB aims to anesthetize the dorsal rami of the spinal nerves, potentially offering a more targeted analgesic effect with fewer side effects.^17^

Although earlier research has demonstrated that SIFIB and L-ESPB can decrease opiate consumption after surgery,^19,20^ no studies compared their efficacy in postoperative pain management for adults having oncologic thigh surgeries under general anesthesia. The study aimed to evaluate the intraoperative and postoperative analgesic effects of SIFIB and L-ESPB in patients who underwent oncologic thigh operations.

## 2. Methodology

This prospective, randomized, controlled, double-blind study involved 75 patients recruited from the National Cancer Institute, Cairo University, Egypt, from June 2022 to September 2024. Every patient undergoing thigh cancer surgery under general anesthesia was screened for eligibility. The study was approved by the institutional review board (AP2203-301-014) and registered at clinicaltrials.gov (ID NCT05393726). Each patient was asked to sign a document indicating his/her fully informed consent.

The study enrolled patients aged 18 to 65 years, of either sex, with a body mass index (BMI) between 20 and 40 kg/m^2^, and an American Society of Anesthesiologists (ASA) physical status of II-III, who planned to have thigh cancer surgery under general anesthesia.

Exclusion criteria were allergies or contraindications to local anesthetics, coagulopathy, posterior thigh mass, participants who used opioids or abused substances (including alcohol) in the past, psychiatric disorders that hinder the ability to perceive and evaluate pain accurately, contraindications to regional anesthesia, and severe renal and hepatic impairment.

## 3. Randomization and blinding

An online randomization program (http://www.randomizer.org) was used to generate a random assignment list, ensuring that participants were allocated randomly, and every patient had a secret code number. Patients were assigned randomly to 3 groups using a 1:1:1 allocation ratio in a parallel manner: the SIFIB group received US-guided SIFIB with 40 mL of 0.25% bupivacaine and 2 mL dexamethasone (4 mg/mL), while the ESPB group received US-guided L-ESPB with an equivalent volume and concentration of the same solution, and the control group received no nerve block. All patients in the 3 groups received the standard perioperative protocol for general analgesia. The blocks were performed after the induction of general anesthesia by an anesthesiologist who did not participate in the other parts of the study. The participants and outcome assessors were blinded to group assignments.

## 4. Anesthetic consideration

In the holding area, eligible patients were examined, taking medical histories and undergoing laboratory assessments, including a complete blood count, coagulation studies, liver and kidney function tests, and an electrocardiogram for all patients. An 18-gauge intravenous (IV) cannula was inserted, and 0.02 mg/kg midazolam was administered. Thirty minutes before the surgical procedure, 7 to 10 mL/kg of IV Ringer's acetate was provided to address fluid deficits. Patients were educated on how to communicate their pain levels using the visual analogue scale (VAS), where 0 represents “no pain” and 10 signifies “the worst possible pain”.

## 5. Intraoperative

In the operating theater, patients were monitored using the standard ASA monitors: pulse oximetry, noninvasive blood pressure measurements, electrocardiogram, and capnography. Then, induction of anesthesia was performed by intravenous propofol (2 mg/kg) and fentanyl (1 μg/kg). Endotracheal intubation was facilitated by atracurium (0.5 mg/kg). Anesthesia was maintained with 1.2% isoflurane in 50% oxygen, with intravenous atracurium (0.1 mg/kg) administered every 20 minutes. Mechanical ventilation was used to maintain end-tidal CO_2_ levels between 35 and 40 mm Hg. Intravenous fentanyl boluses (0.5 µg/kg) were administered if the mean arterial pressure (MAP) or heart rate (HR) exceeded 20% of baseline values.

The blocks were performed using an HFL38X linear multifrequency probe (6–13 MHz) from the portable ultrasound system (SonoSite MTurbo). The blocks were performed under sterile techniques. A mixture of 40 mL of 0.25% bupivacaine and 2 mL of dexamethasone at a concentration of 4 mg/mL was typically administered.

## 6. Ultrasound-guided suprainguinal fascia iliaca block

Patients were in supine position, while an assistant or strong adhesive tape was used to retract any abdominal pannus. An obliquely positioned high-frequency linear US probe was used throughout the procedure; the probe marker was aimed at the midpoint between the xiphoid process and the umbilicus. The anterior superior iliac spine (ASIS) was the site of measurement with the probe. After locating the hyperechoic shadow of the ASIS, the probe was advanced inferomedially from the ASIS following the inguinal ligament's line of descent. The appearance of the sartorius muscle inferolaterally and the internal oblique muscle superomedially created a distinctive superficial “*bow-tie*” or “*hourglass*” shape. Verified by the presence of the hyperechoic ASIS beneath the fascia iliaca and the iliopsoas muscle belly below this hourglass structure, the probe was positioned correctly.

**Figure 1.**
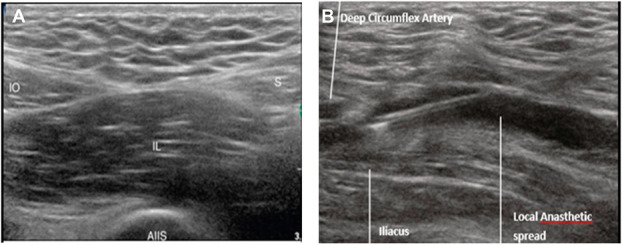
Ultrasound image showing (A) internal oblique (IO) muscle, sartorius (S) muscle, iliacus muscle (IL), anterior inferior iliac spine (AIIS), and (B) separation of internal oblique muscle and the iliacus muscle after injection of local anesthetics.

After this, an 80-mm block needle with a 22-gauge tip was inserted using an in-plane approach, with the inguinal ligament on the lower end and the fascia iliaca on the upper end. A local anesthetic or saline solution of 1 to 2 mL was injected into the hyperechoic fascia iliaca, and its distribution between the more heterogeneous iliacus muscle below was observed to confirm proper spread. The iliacus muscle was hydrodissected from the overlying fascia iliaca after the needle was advanced further into the pocket of local anesthetic in a cephalad direction after confirmation (Figure 1).

## 7. Ultrasound-guided lumbar erector spinae plane block

**Figure 2.**
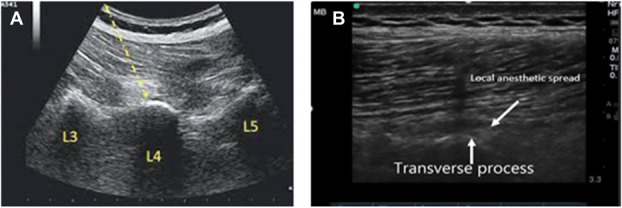
Lumbar erector spinae plane block. (A) An ultrasound image depicting the erector spinae muscle alongside the transverse processes of the lumbar vertebrae, (B) illustrating the distribution of local anesthetic in the intervening space.

Patients were positioned laterally. The 2 iliac crests can be connected by drawing a line, the conventional method for identifying the spinal column, where the fourth lumbar vertebra is located. The next step was to place a low-frequency convex US transducer in the sagittal plane along the midvertebral line. The transducer was subsequently moved laterally, approximately 3.5 to 4 cm from the midline, towards the surgical side to obtain a clear view of the erector spinae muscle and the transverse process.^27^ A 22 G/80 mm block needle was advanced until it touched the transverse process in the out-of-plane procedure. After this, a local anesthetic or 1 to 2 mL of saline was administered to make hydrodissection easier, thereby confirming the accurate placement of the needle (Figure 2).

## 8. Postoperative

After the surgical procedure, neostigmine (0.05 mg/kg) and atropine (0.02 mg/kg) were given to reinstate the remaining neuromuscular blockade. Following the procedure, individuals were moved to the postanesthesia care unit (PACU) and observed until their modified Aldrete score reached or exceeded 9. Subsequently, patients were moved to the ward to receive 1 gm IV acetaminophen and 30 mg ketorolac IV infusion every 8 hours as a part of a multimodal analgesic protocol.

Postoperative pain scores, MAP, and HR were documented at 1, 2, 4, 6, 12, and 24 hours. In cases where the VAS score ≥4, intravenous morphine was given at a dosage of 3 mg; the maximum allowable dose did not exceed 0.3 mg/kg/d. The total morphine administered over 24 hours was documented for each patient. The degree of patient satisfaction at 24 hours postoperative was assessed using a 5-point Likert scale where 1 = extremely dissatisfied, 2 = unsatisfied, 3 = neutral, 4 = satisfied, and 5 = extremely satisfied. Patients used a verbal scale with 4 points to rate the intensity of their nausea and vomiting as none (absence of nausea), mild (nausea without emesis), moderate (a single episode of emesis), or severe (multiple episodes of emesis).

## 9. Outcomes

The primary outcome was postoperative opioid consumption in the first 24 hours. Secondary outcomes were the intraoperative fentanyl consumption, the time of first rescue analgesia, pain VAS scores at rest and during limb movement at 0, 4, 8, 12, and 24 hours postoperatively, patient satisfaction, hemodynamics, and adverse effects.

## 10. Sample size calculation

G*Power software program, version 3.1.9.2 (Universitat Kiel, Germany), was used to determine the required sample size. The means of morphine consumption in SIFIB and L-ESPB were equivalent, with a statistical power of 95% and a type I error of 0.05. Based on the studies of Kumar et al.^19^ and Mostafa et al.,^22^ the mean total morphine consumption in the first 24 hours was 6.95 ± 2.14 mg in SIFIB and 5.0 ± 0.563 mg in L-ESPB, resulting in a final sample size of 21 participants per group. We added 4 cases in each group to overcome dropout; therefore, there were a total of 75 participants.

## 11. Statistical analysis

SPSS v27 (IBM, Chicago, IL) was used for data analysis. The data distribution normality was assessed via the Shapiro–Wilk test and histograms. Normally distributed quantitative data are expressed as mean ± SD and analyzed using ANOVA (F) with Tukey post hoc tests, while nonparametric quantitative data, presented as median and range, were analyzed with the Kruskal–Wallis test, followed by Dunn's test. Categorical variables are presented as frequencies and percentages and were analyzed with the chi-square or Fisher's exact test, and statistical significance was defined as a two-tailed *P*-value <0.05.

## 12. Results

**Figure 3.**
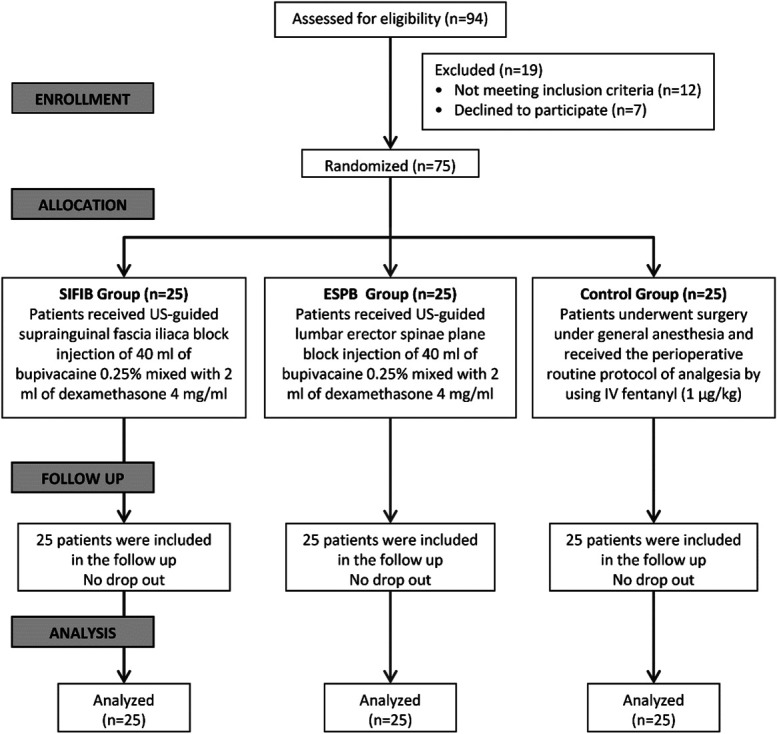
CONSORT diagram of the enrolled patients.

Ninety-four patients underwent eligibility assessments; 12 did not fulfill the requirements for inclusion, and 7 declined to participate. A total of 75 people were recruited, with 25 assigned to each of the 3 groups and included in the statistical analysis and follow-up (Figure 3).

No statistically significant differences were observed between the 3 groups in demographic data or procedure length (Table 1).

**Table 1:** Demographic data and duration of the procedure for the 3 tested groups.

	SIFIB group (n = 25)	ESPB group (n = 25)	Control group (n = 25)	*P*
Age, y	52.3 ± 7.5	48.4 ± 10.9	46.7 ± 11.5	0.140*
Sex				0.675†
Male	15 (60%)	17 (68%)	14 (56%)	
Female	10 (40%)	8 (32%)	11 (44%)	
Weight, kg	92.1 ± 5.3	92.2 ± 6.0	89.7 ± 8.7	0.343*
Height, cm	165 ± 4	165 ± 3	165 ± 5	0.790*
Duration of the procedure, min	159 ± 37	155 ± 57	157 ± 39	0.942*

Data are presented as mean ± SD or frequency (%).

* One-way ANOVA

† χ^2^ test.

During the 24 postoperative hours, only 6 patients (24%) in the SIFIB group and 3 (12%) in the ESPB group required a single dose of 3 mg of morphine. Meanwhile, 20 patients in the control group needed postoperative morphine at a median dose of 8 mg (range: 3–12 mg). The need for morphine was significantly lower in the SIFIB and ESPB groups compared with the control group (*P* < 0.001). However, there was no significant difference between the SIFIB and ESPB groups regarding the need for rescue morphine (*P* = 0.463) (Table 2).

**Table 2:** Number of patients receiving postoperative morphine, intraoperative fentanyl consumption, time to first rescue analgesia, and postoperative complications for the 3 tested groups.

	SIFIB group (n = 25)	ESPB group (n = 25)	Control group (n = 25)	*P*	Post hoc
Number of patients who needed morphine	6 (24%)	3 (12%)	20 (80%)	<0.001^A^*	
Intraoperative fentanyl consumption (µg), mean ± SD	96.0 ± 10.9	94.2 ± 10.7	157.2 ± 32.0	<0.001^B^*	*P*1 = 0.953 *P*2 < 0.001* *P*3 < 0.001*
Time to first rescue analgesia (min), median (range)	330 (180–600)	480 (360–660)	15 (15–180)	<0.001^C^*	*P*1 = 1.000 *P*2 < 0.001* *P*3 = 0.002*
Postoperative complications					
Nausea and vomiting					
Mild	1 (4%)	1 (4%)	3 (12%)	<0.001^A^*	
Moderate	0 (0%)	0 (0%)	5 (20%)		
Severe	0 (0%)	0 (0%)	4 (16%)		
Hypotension	6 (24%)	4 (16%)	2 (8%)	0.304^A^	
Bradycardia	4 (16%)	3 (12%)	1 (4%)	0.375^A^	
Patients' satisfaction					
Extremely satisfied	16 (64%)	14 (56%)	7 (28%)	0.042^A^*	
Satisfied	5 (20%)	6 (24%)	5 (20%)		
Neutral	4 (16%)	5 (20%)	10 (40%)		
Unsatisfied	0 (0%)	0 (0%)	3 (12%)		

Data are presented as frequency (%), mean ± SD, or median (range).

* Significant *P*-value <0.05. A: χ^2^ test, B: one-way ANOVA, C: Kruskal–Wallis test. *P*1: *P*-value between SIFIB and ESPB groups, *P*2: *P*-value between SIFIB and control groups, *P*3: *P*-value between ESPB and control groups.

Both SIFIB and ESPB groups showed significantly lower intraoperative fentanyl consumption (96.0 ± 10.9 μg and 94.2 ± 10.67 μg, respectively), compared with the control group (157.2 ± 32.0 μg) (*P* < 0.001). However, the intraoperative fentanyl consumption was insignificantly different between SIFIB and ESPB groups (*P* = 0.953) (Table 2).

Time to first rescue analgesia was significantly longer in the SIFIB and ESPB groups compared with the control group (*P* < 0.001). However, there was no significant difference between the ESPB and the SIFIB groups (*P* = 1.000) (Table 2).

**Figure 4.**
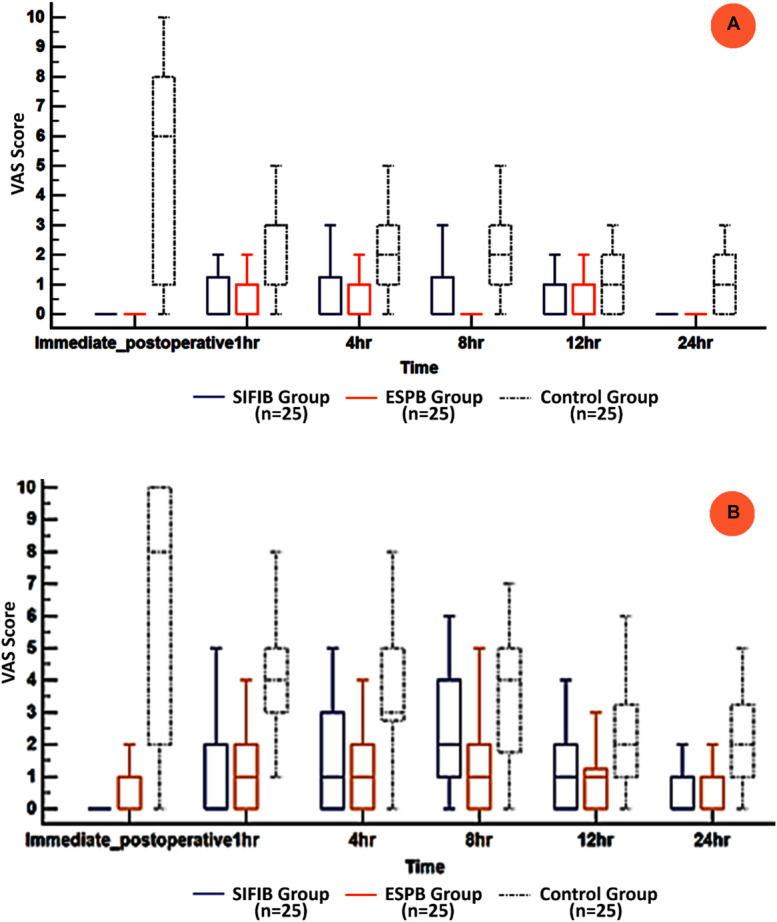
(A) VAS at rest and (B) VAS during limb movement in the 3 groups. Data are presented as median (IQR), and the groups were compared with the Kruskal–Wallis test.

Visual analogue scale (VAS) scores at rest and during movement were significantly lower in the SIFIB and ESPB groups at all times of postoperative measurement compared with the control group and showed no significant difference between the SIFIB and ESPB groups. Pain scores at rest decreased by 60% at 1 minute postoperatively in both the SIFIB and ESPB groups compared to the control group, while pain scores during limb movement were reduced by 80% in these groups (Figure 4).

Both SIFIB and ESPB groups reported higher patient satisfaction compared with the control group (*P* = 0.042) and lower frequency of postoperative nausea and vomiting (PONV). Only one patient in the SIFIB and ESPB groups had PONV compared with 12 (48%) in the control group (*P* < 0.001). Hypotension and bradycardia showed no meaningful differences between the 3 cohorts. Respiratory depression was not observed in any patient (Table 2).

**Figure 5.**
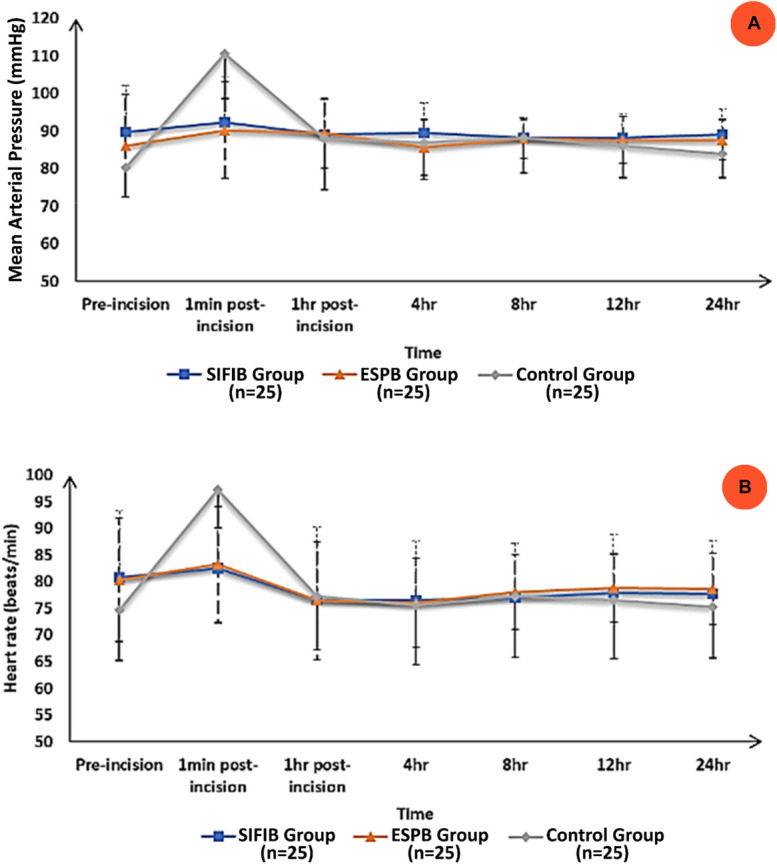
Hemodynamics data preincision and during the 24 hours postoperatively: (A) Mean blood pressure and (B) heart rate measurements in the 3 groups. Data are presented as mean ± SD and the groups were compared with a one-way ANOVA test.

There was no significant difference between groups in the MAP before incision. One minute after surgical incision, MAP was significantly lower in the SIFIB and ESPB groups compared with the control group (*P* = 0.003). Thereafter, there was no significant difference in the MAP up to 24 hours postoperatively between the 3 groups (Figure 5A).

Heart rate measurements showed similar results. There was no significant difference between groups in HR before incision, and one minute after incision, HR was significantly lower in the SIFIB and ESPB groups compared with the control group (*P* = 0.009). Then, there was no significant difference between the 3 groups in the HR up to 24 hours postoperatively (Figure 5B).

## 13. Discussion

The necessity for efficient analgesia during the perioperative phase of major lower-limb surgery has inspired concern in regional anesthesia. Regional anesthetic of the lower limbs, as a part of a multimodal analgesic approach, may offer significant benefits, including reduced opioid consumption, decreased hospital duration, enhanced patient satisfaction, and improved functional results.^16,24^ However, the best regional analgesic technique has yet to be identified. In the current study, we have selected 2 relatively novel techniques to administer analgesia to patients with thigh cancers undergoing limb salvage surgeries.

The study demonstrated that SIFIB and L-ESPB were associated with reduced perioperative opioids and pain intensity. Few patients in the 2 groups requested morphine during the postoperative period compared with 80% of the control group. The time to the first request for analgesia was significantly longer in the 2 groups than in the controls. Similarly, both SIFIB and ESPB groups showed significantly lower intraoperative fentanyl consumption compared with the control group. Pain scores at rest and during movement were significantly lower in the SIFIB and ESPB groups than in the control group. The 2 techniques displayed comparable analgesic profiles and hemodynamic stability. The 2 regional block techniques saved the patients from the increase in heart rate and MAP in response to surgical incision, and the opioid-induced nausea and vomiting.

Studies comparing the 2 regional techniques, SIFIB and L-ESPB, are scarce, especially in cases of lower-limb surgery. Consistent with the present findings, Flaviano et al.^10^ found that lumbar ESPB and SIFIB provided comparable reduction of pain intensity and morphine consumption in patients undergoing total hip arthroplasty. In patients undergoing lower-extremity surgeries, SIFIB alone or combined with a sciatic block was described as a valid alternative to lumbar plexus block.^13^ However, SIFIB was less effective in terms of pain reduction and opioid sparing than pericapsular nerve group block in patients undergoing hip and proximal femur surgery.^14^

suprainguinal fascia iliaca block is recognized as a good analgesic technique. suprainguinal fascia iliaca block surpasses placebo in reducing pain and promoting early postoperative movement in patients undergoing general or spinal anesthesia.^6^ It is particularly beneficial in orthopedic contexts involving hip or femoral surgery. The 2021 PROSPECT guidelines endorse SIFIB as the optimal peripheral block for hip arthroplasty.^2^

We believe that SIBIF efficacy stems from blocking the innervation of the anterior and medial thigh, from the femoral, lateral femoral cutaneous, and obturator nerves,^8^ situated in the fascia iliaca compartment.^30^ suprainguinal fascia iliaca block is a modification of the fascia iliaca compartment block (FICB), in which local anesthetic (LA) is administered superior to the inguinal ligament to obstruct the femoral nerve proximally,^15^ promoting cranial diffusion of LA within the fascia iliaca compartment, thereby obstructing the femoral nerve proximal to the origins of the high articular branches.^25,31^

In this study, we administered a LA volume of 40 mL to achieve the blocking of the 3 nerves, including the obturator nerve (ON). There is no consensus about the ideal volume of LA for SIFIB to block the femoral and lateral cutaneous femoral nerves. Besides, the volume of LA necessary to simultaneously block ON remains contentious. Bravo et al.^4^ indicated that 40 mL of 0.25% levobupivacaine can inhibit both the sensory and motor functions of ON. Blocking the 3 nerves was confirmed in a recent cadaver study.^32^ Therefore, it appears that the LA volume is crucial to the efficacy of SIFIB, and the results of the current study may be influenced by using a different volume of LA.

This study revealed that L-ESPB exhibited analgesic and opioid-sparing efficacy equivalent to that of SIFIB. This procedure is intended to block the innervation of the lower limbs formed within the psoas muscle, derived from the anterior rami of T12-L4.^8^ The lumbar plexus block is the predominant analgesic method for knee, femoral shaft, or hip procedures.^5,12,23,26^ In L-ESPB, the LA extends anteriorly within the paravertebral region, primarily around the psoas muscle and lumbar plexus.^18^ A prospective trial of total hip replacement showed that L-ESPB significantly reduced postoperative pain and opioid usage and maintained stable intraoperative hemodynamics.^20^

In this study, LA in a volume of 40 mL was injected at the level of the fourth lumbar vertebra. Tulgar et al.^28^ indicated that L-ESPB is effective for postoperative analgesia in hip/femur surgery, with contrast material dispersing to T12-S1 on CT assessment. The dissemination of LA between the T12 and S1 dermatomes was observed with the administration of 30 to 40 mL of LA.^1^ The craniocaudal distribution of a 40 mL LA mixture provided analgesia from T10 to S2 with a significant reduction of the VAS score 30 minutes after block in patients suffering from herpes zoster.^1^

Studying has some limitations. The study's generalizability may be limited because of its single-center design. The study compared SIFIB and ESPB across different types of thigh surgeries, which could introduce variability in outcomes based on the specific surgical procedure. It can be argued that the decision not to insert a catheter to extend the analgesic effect of LA was deliberate; the study design specifically aimed to avoid catheter placement to minimize patient discomfort and the risk of complications such as migration. The study did not assess the quality of sleep or longer-term postoperative outcomes, which could provide additional insights into the effectiveness of the analgesic techniques. The small number of cases requesting postoperative rescue morphine did not allow robust conclusions about the time to first request rescue analgesia after regional blocks. We did not test the dermatomal coverage of the used blocks.

## 14. Conclusion

In patients undergoing oncologic thigh surgery, ultrasound-guided suprainguinal fascia iliaca block (SIFIB) and lumbar erector spinae plane block (L-ESPB) are comparable, effective, and safe regional analgesic alternatives. Both techniques significantly reduce pain scores and overall opioid consumption, and delay the time to first rescue analgesia while improving patient satisfaction and minimizing postoperative nausea and vomiting.

## Disclosures

The authors have no conflict of interest to declare.
